# Blood glucose variability in early-onset adrenocorticotropic hormone deficiency induced by immune checkpoint inhibitor therapy with continuous blood glucose monitoring: a case report

**DOI:** 10.1007/s13340-025-00870-6

**Published:** 2026-01-12

**Authors:** Yuta Nanao, Gentaro Egusa, Ryuta Baba, Takaya Kodama, Tsuguka Matsuda, Gaku Nagano, Haruya Ohno

**Affiliations:** 1 Department of Diabetes, Metabolism and Endocrinology, Miyoshi Central Hospital, Higashisakayacho, Hiroshima, Miyoshi, 728–8502 Japan; 2 Department of Endocrinology and Diabetes, Hiroshima University Hospital, 1–2-3 Kasumi, Hiroshima, Minami-ku, 734–8551 Japan

**Keywords:** Immune checkpoint inhibitors (ICIs), Immune-related adverse events (irAEs), Hypopituitarism, Adrenal insufficiency, Nocturnal hypoglycemia, Continuous glucose monitoring device (CGM)

## Abstract

Early diagnosis and treatment of immune-related adverse events (irAEs) associated with immune checkpoint inhibitors (ICIs) are essential because they directly impact patient quality of life. This report describes the case of an 85-year-old woman with type 2 diabetes on insulin therapy, whose glycemic fluctuations became highly unstable following irAE development. During treatment for refractory hepatocellular carcinoma with tremelimumab and durvalumab, she developed hyperglycemia and was hospitalized. Endogenous insulin secretion remained intact, and hyperglycemia improved after admission. Continuous glucose monitoring (CGM) revealed nocturnal and early-morning hypoglycemia from the fourth day of admission. Insulin requirements were tapered off; however, persistent anorexia and dyspnea led to the diagnosis of hypopituitarism through endocrine testing. For patients with diabetes who experience abnormal blood glucose fluctuations after ICI therapy, clinicians should monitor changes in endogenous insulin secretion and consider the possibility of hypoadrenocorticism. CGM may be valuable for detecting these endocrine abnormalities.

## Introduction

Immune checkpoint inhibitors (ICIs) have become widely used as an essential treatment option for cancer, with increasing application to improve patient prognosis. However, these drugs have gained attention due to their immune-related adverse events (irAEs), which affect multiple organs and significantly impact patient quality of life [1].

Endocrine disorders caused by irAEs include type 1 diabetes mellitus, thyrotoxicosis, and hypoadrenocorticism, leading to abnormal blood glucose levels such as hyperglycemia and hypoglycemia. A sudden decrease in insulin secretion results in hyperglycemia and diabetic ketoacidosis, whereas nocturnal and early-morning hypoglycemia may be an initial symptom of hypoadrenocorticism [2]. However, diagnosing hypoadrenocorticism may be delayed for patients already receiving insulin therapy or sulfonylurea (SU) drugs.

Herein, we present a case of a patient with diabetes in whom blood glucose fluctuations associated with ICI-induced hypopituitarism were observed using continuous glucose monitoring (CGM). Nocturnal and early-morning hypoglycemia were detected, leading to the diagnosis of hypopituitarism and treatment initiation.

## Case presentation

The patient was an 85-year-old woman diagnosed with type 2 diabetes at the age of 42 years. Prior to admission, her diabetes treatment consisted of linagliptin (5 mg/day), mitiglinide (20 mg/day), and 4 units of insulin degludec/insulin aspart (IDeg/Asp) injections in the morning. Glycoalbumin levels ranged from 22 to 26%. She had no history of endocrine disorders, although two sisters and one brother had type 2 diabetes, without family history of malignancy or endocrine disease.

**Fig. 1:**
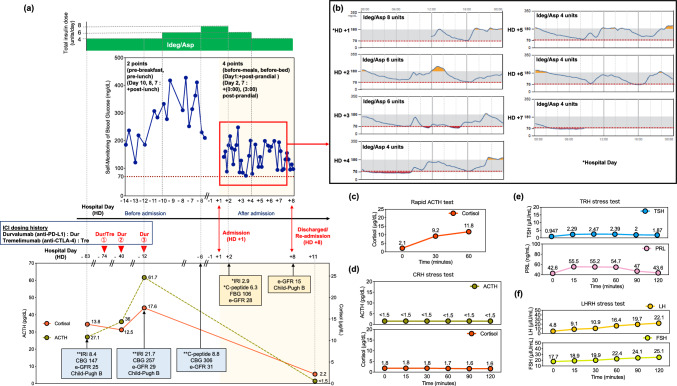
**a** Progression of fasting blood glucose self-monitoring, insulin administration, ACTH and cortisol levels, and ICI dosing history by Hospital Day (HD). The horizontal axis represents HD was defined as the day of admission (HD + 1), with preceding days labeled as HD − 1, HD − 6, HD − 7, etc. Days prior to HD + 1 (i.e., HD -1 and earlier) were considered before admission, whereas HD + 1 and subsequent days were considered after admission. The vertical axis (from top to bottom) indicates the insulin units, fasting blood glucose, ICI dosing history (red triangle), ACTH and cortisol levels. The dark blue line represents the trend of fasting blood glucose levels obtained from self-monitoring. Data from HD − 5 to − 1 are missing. The green line shows the ACTH levels, whereas the orange line represents the cortisol levels. ACTH and cortisol were measured only on HD − 83, − 40, − 12 and + 11. Since they were not measured on other days, their changes are represented with dotted lines. The unit of IRI is μU/mL. The unit of CPR is ng/mL. The unit of fasting blood glucose (FBG) and casual blood glucose (CBG) is mg/dL. The unit of e-GFR is mL/min/1.73 m^2^.** b** Depicts CGM trends from HD + 1 to + 7.** c** Rapid ACTH test.** d** CRH stimulation test.** e** TRH stimulation test.** f** LHRH stimulation test. (**c**–**f**) present the results of the rapid ACTH test on HD + 12 and the CRH, TRH, and LHRH stress test results on HD + 16. The horizontal axis of each graph represents the elapsed time of the test, and the vertical axis indicates the corresponding evaluation parameters. *fasting; **casual; ACTH, adrenocorticotropic hormone; CBG, casual blood glucose; CGM, continuous glucose monitoring; CRH, corticotropin-releasing hormone; eGFR, estimated glomerular filtration rate; FBG, fasting blood glucose; ICI, immune checkpoint inhibitor; IDeg/Asp, insulin degludec/insulin aspart; IRI, immunoreactive insulin; LHRH, luteinizing hormone-releasing hormone; TRH, thyrotropin-releasing hormone

Based on hepatitis B virus–related cirrhosis, the patient was diagnosed with hepatocellular carcinoma at age 78 years; she underwent hepatic arterial embolization at ages 84 and 85 years. She received tremelimumab (anti-CTLA-4 antibody) and durvalumab (anti-PD-1 antibody) on Hospital Day (HD) − 74, followed by additional durvalumab on HD − 40 and − 12 (Fig. 1a-lower panel). The patient had a Child–Pugh class B (score 7) on HD -83 before ICI therapy (Fig. 1a-lower panel).

Immediately after the third dose of durvalumab, on HD − 11, her blood glucose levels remained persistently > 300 mg/dL. She was hospitalized for further evaluation and treatment for suspected irAEs-induced type 1 diabetes.

At admission, the patient was 146 cm tall, weighed 51 kg (body mass index 23.9 kg/m^2^), had clear consciousness, temperature of 36.5 ℃, blood pressure of 115/48 mmHg, and pulse of 70 beats/min.

The blood test results on HD − 40 showed levels of adrenocorticotropic hormone (ACTH) 36.0 pg/mL and cortisol 12.5 μg/dL (not measured immediately after waking). On HD − 12, the ACTH level was 61.7 pg/mL and the cortisol level was 17.6 μg/dL (Fig. 1a-lower panel). The blood test results on the day after admission showed a fasting blood glucose level of 106 mg/dL, glycoalbumin level of 28.8%, C-peptide level of 6.3 ng/mL, and anti-GAD antibodies below 5.0 U/mL (Table 1a). The trends of insulin, plasma C-peptide, and blood glucose levels are displayed in (Fig. 1a-lower panel). The fasting plasma C-peptide level on HD + 2 was high (6.3 ng/mL); however, it was comparable to the level measured on HD − 6 (8.8 ng/mL). This result was considered to be influenced by chronic kidney disease and a decreased estimated glomerular filtration rate (Fig. 1a-lower panel). At this stage, hypoadrenocorticism was not suspected.

**Table 1:** Laboratory data on admission

(a) HD + 2					
Hematology and biochemistry		Units	Biochemistry		Units
White blood cell	3400	/μL	γ-GTP	32	U/L
Neutrophil	73.3	%	Amylase	53	U/L
Lymphocyte	13.8	%	Total protein	6.0	g/dL
Monocyte	9.7	%	Albumin	2.7	g/dL
Eosinophil	2.9	%	Blood urea nitrogen	46.3	mg/dL
Red blood cell	2.84 × 10^4^	/μL	Creatinine	1.4	mg/dL
Hemoglobin	9.0	g/dL	e-GFR	28.0	mL/min/1.73m^2^
Hematocrit	29.9	%	Uric acid	3.8	mg/dL
MCV	105.3	fL	CRP	0.7	mg/dL
Platelet	8.4 × 10^4^	/μL	Triglyceride	113	mg/dL
Sodium (Na)	144	mEq/L	HDL-cholesterol	53	mg/dL
Potassium (K)	4.6	mEq/L	LDL-cholesterol	89	mg/dL
Chloride (Cl)	109	mEq/L	Cholesterol	161	mg/dL
Corrected calcium	9.7	mg/dL	Glycoalbumin	28.8	%
AST	22	U/L	Plasma glucose	106	mg/dL
ALT	16	U/L	GAD Ab	< 5.0	U/mL
LDH	321	U/L	CPR	6.3	ng/mL
ALP	129	U/L	IRI	2.9	μU/mL
(b) HD + 8					
Hematology and biochemistry		Units	Biochemistry		Units
White blood cell	3230	/μL	LDH	244	U/L
Neutrophil	67.2	%	ALP	150	U/L
Lymphocyte	16.7	%	γ-GTP	38	U/L
Monocyte	11.8	%	Amylase	46	U/L
Eosinophil	4.0	%	Pancreas-Amylase	17	U/L
Red blood cell	2.97 × 10^4^	/μL	Total bilirubin	0.3	mg/dL
Hemoglobin	9.8	g/dL	Direct bilirubin	< 0.1	mg/dL
Hematocrit	31.2	%	Total protein	6.0	g/dL
MCV	105.1	fL	Albumin	2.3	g/dL
Platelet	12.1 × 10^4^	/μL	Blood urea nitrogen	95.1	mg/dL
Na	139	mEq/L	Creatinine	2.5	mg/dL
K	5.3	mEq/L	e-GFR	15.0	mL/min/1.73m^2^
Cl	108	mEq/L	Uric acid	4.9	mg/dL
Corrected calcium	9.9	mg/dL	Plasma glucose	109.0	mg/dL
AST	17	U/L	CRP	5.6	mg/dL
ALT	12	U/L	Procalcitonin	0.2	ng/mL
(c) HD + 15					
Endocrinology		Units	Endocrinology		Units
GH	10.2	ng/mL	LH	10.1	μIU/mL
Somatomedin C	30.0	ng/mL	FSH	23.6	μIU/mL
PRL	15.9	ng/mL	Estradiol	9.0	pg/mL
TSH	1.1	μIU/mL	AVP	4.3	pg/mL
FT3	1.9	pg/mL	PRA	8.6	ng/mL/hr
FT4	1.2	ng/mL	PAC	< 0.4	ng/dL

MCV : Mean corpuscular volume, AST : Aspartate aminotransferase, ALT : Alanine aminotransferase, LDH : Lactate dehydrogenase, ALP : Alkaline phosphatase, γ-GTP : Gamma-glutamyl transpeptidase, e-GFR : estimated Glomerular Filtration Rate, GAD Ab : Anti-glutamic acid decarboxylase antibodies, CPR : C-peptide reactivity, IRI : Immunoreactive insulin, CRP : C-reactive protein, GH : Growth Hormone, PRL : Prolactin, TSH : Thyroid stimulating hormone, FT4 : Free thyroxine, FT3 : Free triiodothyronine,LH : Luteinizing hormone, FSH : Follicle-stimulating hormone, ACTH : Adrenocorticotropic hormone, AVP : Arginine vasopressin, PRA : Plasma renin activity, PAC : Aldosterone (CLEIA)

A 1400 kcal/day diet was initiated, and the IDeg/Asp dosage was adjusted according to blood glucose fluctuations (Fig. 1a-upper panel).

On the day of admission, the patient was fitted with CGM (FreeStyle Libre pro®, Abbott Laboratories, Chicago, IL, USA). Hyperglycemia improved immediately after admission, leading to a reduction in IDeg/Asp from 8 to 6 units. Figure 1b illustrates the blood glucose trends observed with CGM.

On HD + 2, early-morning fasting blood glucose decreased to 114 mg/dL, without postprandial hyperglycemia observed in CGM. After admission, the patient was able to perform the insulin self-injection technique without error.

During hospitalization, urinary C-peptide was assessed twice using a 24-h urine storage test, averaging 26.4 μg/day.

On HD + 4, nocturnal and early-morning hypoglycemia (observed in patients with CGM) led to a reduction in IDeg/Asp from 6 to 4 units (Fig. 1b). Blood glucose fluctuations were assessed using both CGM and frequent self-monitoring of blood glucose (SMBG); no nocturnal hypoglycemia was confirmed by HD + 7 (Fig. 1a-upper panel). Eight days later (on HD + 8), the patient was discharged.

However, on the night of discharge, she was rushed to our hospital with dyspnea. During transport, her consciousness was clear, temperature 36.5 ℃, blood pressure 115/48 mmHg, pulse 70 beats/min, respiratory rate 16 breaths/min, and SpO₂ 98% (room air). Blood tests (Table 1b) showed no white blood cell count elevation, although the C-reactive protein level was elevated, and a computed tomography scan revealed infiltrative shadows mainly in the lower lobes of the right lung. On the same day, hepatic reserve was assessed and a Child–Pugh score of 7 (class B) was determined, which did not change from the pre-ICI evaluation (Fig. 1a-lower panel).

Pneumonia was suspected, and the patient was admitted to our hospital. After readmission (on HD + 9), dyspnea improved; however, anorexia persisted, and blood glucose level remained low. Owing to persistent anorexia, we suspected an endocrine abnormality and performed a close examination on HD + 11.

Early-morning fasting blood tests indicated low ACTH and cortisol (Fig. 1a-lower panel). A rapid ACTH test on HD + 12 revealed peak cortisol values below 18 μg/dL after a 0.25 mg tetracosactide load (Fig. 1c). Free cortisol from a 24-h urine sample was below 8.0 μg/day. Endocrinological evaluation revealed low free triiodothyronine (FT3), elevated growth hormone (GH), reduced estradiol (E2), and decreased (plasma aldosterone concentration) PAC (Table 1c). A head magnetic resonance imaging on HD + 15 revealed an empty sella (data not shown). Corticotropin-releasing hormone (CRH), luteinizing hormone-releasing hormone (LHRH), and thyrotropin-releasing hormone (TRH) stress tests on HD + 16 showed peak ACTH, thyroid stimulating hormone, luteinizing hormone, and follicle-stimulating hormone values below reference values after administration of 100 μg CRH, 0.2 mg TRH, and 0.1 mg LHRH, LHRH and TRH stress tests (Fig. 1d–f). The insulin tolerance test was not performed due to the patient's advanced age and poor general condition.

Based on these findings, ICI-induced hypopituitarism and secondary hypoadrenocorticism were diagnosed.

On HD + 16, due to severe malaise, intravenous hydrocortisone (150 mg/day) was initiated and tapered based on symptoms. By HD + 23, oral hydrocortisone (40 mg/day) was introduced, with dose adjustments as needed. Since her blood glucose levels rose with the initiation of hydrocortisone, on HD + 17 we switched the patient’s diabetes treatment from IDeg/Asp to a combination of basal insulin degludec and regular insulin to allow for more precise dosage adjustment. Early morning hypoglycemia was subsequently observed, and insulin degludec was discontinued on HD + 34.

The patient was discharged on HD + 47, oral hydrocortisone (30 mg/day) was continued, and diabetes was managed with linagliptin (5 mg/day) and repaglinide (0.75 mg/day).

## Discussion

IrAEs associated with dysglycemia following ICI therapy include type 1 diabetes, hypopituitarism, thyrotoxicosis, and primary adrenal insufficiency [1].

Type 1 diabetes often presents with diabetic ketoacidosis-like symptoms, such as vomiting, thirst, polydipsia, polyuria, and impaired consciousness [3]. In contrast, hypoadrenocorticism may first manifest as nocturnal or early-morning hypoglycemia. Symptoms of adrenocortical hypofunction are diverse and nonspecific, including fatigue, weakness, anorexia, weight loss, gastrointestinal symptoms, hypotension, mental disturbances, fever, and hypoglycemia, particularly at night [2]. Other symptoms include arthralgia, headache, and visual field disturbances [4]. Cortisol is associated with insulin resistance because it decreases insulin sensitivity in the liver and in the extra-liver compartment [5]. Cortisol secretion typically peaks at night and early morning; however, patients with secondary hypoadrenocorticism due to ACTH deficiency are predisposed to late-night or early-morning hypoglycemia because of insufficient cortisol secretion [6]. Fatigue, including hypoglycemic symptoms, often resembles the effects of malignant tumors or anticancer drugs, leading to delayed recognition of hypoadrenocorticism onset [7].

The onset of dysglycemia-related irAEs varies depending on the disease and ICI type. Type 1 diabetes typically develops after a median of 4.5 courses (95% confidence interval [CI]: 1–17 courses) [8] with anti-PD-1 antibodies and 2.7 courses (95% CI: 1–5 courses) with anti-PD-L1 + anti-CTLA-4) [9].

Hypopituitarism onset has been reported at 8–16 weeks (95% CI) with anti-PD-1 antibodies [10], 0–48 weeks (95% CI) with anti-PD-L1 antibodies [10], and 4–10 weeks (95% CI) with anti-CTLA-4 antibodies [10]. In this case, the disease onset occurred approximately 10 weeks after the first administration of tremelimumab (anti-CTLA-4 antibody) and durvalumab (anti-PD-L1 antibody).

Initially, type 1 diabetes was suspected as the primary irAE; however, hypoadrenocorticism was ultimately diagnosed. Before admission, the patient was hyperglycemic, although blood glucose levels stabilized upon hospitalization. Despite subsequent insulin dose reduction, CGM detected nocturnal and early-morning hypoglycemia. The patient initially presented with hyperglycemia prior to admission during ICI therapy, which raised concern about new-onset type 1 diabetes due to impaired insulin secretion. However, plasma and urinary C-peptide levels measured before admission and after hospitalization did not support insulin depletion. The early-morning fasting hypoglycemia observed during CGM after admission was considered to be influenced by several factors: delayed insulin clearance because of impaired renal function, reduced hepatic glucose production related to cirrhosis, and improved insulin sensitivity. Although hepatic and renal dysfunction were present and may have contributed, no significant changes in liver or kidney function occurred before or after admission, making them unlikely primary causes (Fig. 1a-lower panel). Improved insulin sensitivity was attributed to the resolution of glucotoxicity following insulin dose escalation, as well as the diminished effects of insulin-antagonistic hormones such as glucagon and cortisol. Outpatient insulin therapy had been maintained at 4 units per day until 10 days prior to admission, which then increased to 6–8 units per day; however, after admission, the dosage was promptly tapered back to 4 units, representing a relatively low overall insulin requirement (Fig. 1a). Although glucagon levels were not measured, cortisol deficiency may have suppressed the insulin-antagonistic action of glucagon. Given that cortisol plays a key role in promoting insulin resistance, the observed nocturnal and early-morning hypoglycemia was suggestive of hypoadrenalism onset.

Diagnosing hypopituitarism early based solely on nonspecific symptoms such as anorexia is challenging. However, identifying characteristic blood glucose fluctuations, as in this case, may facilitate earlier recognition of adrenal insufficiency.

Akturk et al. reported that measuring blood glucose every 2 weeks in an outpatient setting is ineffective in predicting hyperglycemia due to reduced insulin secretion in irAEs [11].

This report shows that compared to 2- or 4-point SMBG, CGM was more useful in detecting nocturnal and early-morning hypoglycemia (Fig. 1a-upper panel, 1b). Although discrepancies may exist between actual blood glucose levels and CGM readings, CGM has a significant advantage due to its ability to capture glycemic fluctuations during periods such as sleep and early morning, which are difficult to assess using SMBG. The use of CGM, in addition to self-monitoring of blood glucose, may aid in the early diagnosis of both type 1 diabetes and hypoglycemia caused by hypoadrenocorticism.

As new ICIs are currently being developed and introduced into clinical practice, the incidence of irAEs is also increasing; thus, highlighting the need for predictive markers. Genotypes and autoantibodies have been investigated as predictive factors [12]. However, to date, no biomarkers have been validated as predictors of irAEs in asymptomatic patients treated with ICIs [13]. Recently, transient elevations in ACTH and cortisol, indicative of pituitary inflammation, have been reported before the onset of ICI-induced hypopituitarism [14, 15].

From HD − 83 to − 40, the patient's ACTH and cortisol levels were approximately 27.1–36 pg/mL and 12.5–13.8 μg/dL, respectively. However, on HD − 12, the ACTH level increased to 61 pg/mL, and the cortisol level rose to 17.6 μg/dL, indicating a transient elevated ACTH trend. On that same day, the patient also presented with hyperinsulinemia, a casual blood glucose level of 257 mg/dL, and an immunoreactive insulin level of 21.7 μU/mL. On HD + 2, the fasting blood glucose was 106 mg/dL and the immunoreactive insulin level was 2.9 μU/mL. Despite the gradual reduction in the insulin dosage after admission, CGM continued to show nocturnal hypoglycemia (Fig. 1a-upper panel and b). On HD + 11, the ACTH level was below detection sensitivity, and the cortisol level had decreased to 2.2 μg/dL (Fig. 1a- lower panel). In this case, ACTH was also elevated on HD − 12, although it was markedly decreased on HD + 11 when hypopituitarism was suspected. This transition suggests that the patient may have developed pituitary inflammation immediately after the third cycle of durvalumab and subsequently transitioned to hypopituitarism.

Blood glucose fluctuations before and after admission shifted from hyperglycemia to hypoglycemia, corresponding to changes in the ACTH and cortisol levels. Although precise evaluation was challenging due to ongoing insulin therapy and casual blood tests, the endogenous insulin levels appeared to vary in parallel with the cortisol levels. On HD − 12, the elevated insulin level may have resulted from increased insulin resistance due to transient hypercortisolemia. After admission, a decrease in insulin levels and improved glycemic control were observed, suggesting enhanced insulin sensitivity. Considering these results together, the hyperglycemia observed prior to admission may have indicated cortisol-induced insulin resistance, whereas the nocturnal hypoglycemia after admission may have been a consequence of reduced cortisol secretion and subsequent improvement in insulin sensitivity.

Symptoms of hypoadrenocorticism due to irAEs may not always align with ACTH and cortisol levels, emphasizing the need to assess cortisol insufficiency using additional clinical findings. This patient experienced appetite loss before admission; however, blood tests showed normal cortisol levels, without electrolyte abnormalities (sodium or potassium) or elevated eosinophil fractions.

In addition, this patient had chronic fatigue, potentially due to anticancer drug therapy that further complicated the diagnosis, making hypoadrenocorticism an unlikely initial differential diagnosis upon the first admission in this case. Although it is difficult to diagnose hypopituitarism by atypical symptoms such as anorexia and fatigue, by focusing on both ACTH changes and blood glucose fluctuations, hypopituitarism could have been diagnosed early in the course of the disease.

In conclusion, a sudden rise in ACTH following ICI therapy may indicate pre-pituitary inflammation, which can precede hypopituitarism. Hypoglycemia occurring at night or early morning hours after ICI treatment may be a symptom of hypopituitarism, warranting careful monitoring. CGM may facilitate early identification of hypoglycemia, allowing timely diagnosis and intervention. Early recognition of hypopituitarism based on blood glucose trends and ACTH fluctuations may improve patient outcomes and quality of life.
